# Identification and Exploration of Pyroptosis-Related Genes in Macrophage Cells Reveal Necrotizing Enterocolitis Heterogeneity Through Single-Cell and Bulk-Sequencing

**DOI:** 10.3390/ijms26094036

**Published:** 2025-04-24

**Authors:** Peipei Zhang, Ying Li, Panpan Xu, Peicen Zou, Sihan Sheng, Ruiqi Xiao, Pu Xu, Ying Chen, Yue Du, Lishuang Ma, Yajuan Wang

**Affiliations:** 1 Capital Institute of Pediatrics, Chinese Academy of Medical Sciences & Peking Union Medical College, Beijing 100730, China; zpp95@outlook.com (P.Z.); ey_xpp@student.pumc.edu.cn (P.X.); zou_peicen@sina.com (P.Z.); b2024032016@student.pumc.edu.cn (S.S.); xupuxp@126.com (P.X.); 2 Department of Neonatology, Capital Center for Children’s Health, Capital Medical University, No. 2 Yabao Road, Chaoyang District, Beijing 100020, China; jizhenly@163.com (Y.L.); nhcy521@126.com (Y.C.); dy623877932@163.com (Y.D.); 3 Capital Institute of Pediatrics, Beijing 100020, China; ruiqixiao29@163.com; 4 Department of Neonatology Surgery, Capital Center for Children’s Health, Capital Medical University, No. 2 Yabao Road, Chaoyang District, Beijing 100020, China

**Keywords:** necrotizing enterocolitis, pyroptosis, macrophage, TREM1, bioinformatics

## Abstract

Necrotizing enterocolitis (NEC) is an acute intestine dysfunction intestinal disorder characterized by inflammation and cell death, including pyroptosis. Previous studies have implicated pyroptosis, particularly via NLRP3 inflammatory activation, and contribute to the development of NEC. However, the genetic and molecular mechanisms underlying pyroptosis in NEC pathogenesis and sequelae remain unclear. Our study aimed to identify the pyroptosis-related cell populations and genes and explore potential therapeutic targets. Single-cell RNA sequencing (scRNA-seq) data were analyzed to identify the cell populations related to NEC and pyroptosis. Weighted gene correlation network analysis (WGCNA) of bulk RNA-seq was performed to identify gene modules associate with pyroptosis. Cell–cell communication was employed to investigate intercellular signaling networks. Gene Set Enrichment Analysis (GSEA) was conducted to compare the pathways enriched in the high and low TREM1-expressing subgroups. Immunofluorescence staining was performed to detect the TREM1^+^CD163^+^ macrophages in the intestines. PCR and Western blot were performed to detect the expression of mRNA and proteins in the intestine tissues and cells. scRNA-seq analysis revealed increased macrophage abundance in NEC, with one macrophage cluster (cluster 4) exhibiting a markedly elevated pyroptosis score. WGCNA identified a gene module (MEbrown) that positively correlated with pyroptosis. Five genes (*TREM1*, *TLN1*, *NOTCH2*, *MPZL1*, and *ADA*) within this module were identified as potential diagnostic markers of pyroptosis. Furthermore, we identified a novel macrophage subpopulation, *TREM1^+^CD163^+^*, in NEC. Cell–cell communication analysis suggested that *TREM1^+^CD163^+^* macrophages interact with other cells primarily through the NAMPT/ITGA5/ITGB1 and CCL3/CCR1 pathways. GSEA revealed a significant association between high TREM1 expression and pathways related to pyroptosis, cell proliferation, and inflammation. In vivo and in vitro experiments confirmed an increase in *TREM1^+^CD163^+^* macrophages in NEC-affected intestines. TREM1 inhibition in THP-1 cells significantly reduced the expression of pro-inflammatory cytokines and pyroptosis-related genes and proteins. We identified the *TREM1^+^CD163+* macrophage population that plays a crucial role in pyroptosis during NEC progression. Our findings elucidate the biological functions and molecular mechanisms of TREM1, demonstrating its upregulation in vivo and pro-pyroptosis effects in vitro. These insights advance our understanding of the role of pyroptosis in NEC pathogenesis and suggest TREM1 is a potential therapeutic target for NEC.

## 1. Introduction

Necrotizing enterocolitis (NEC) is an acute inflammatory disease characterized by intestinal necrosis. In premature infants, the incidence of NEC can reach up to 10%, while in preterm infants with extremely low birth weights, the incidence rises to an exceptionally high 30–50% [1]. Despite extensive research, NEC pathogenesis remains unclear. Major risk factors include premature birth, intestinal dysbiosis, formula feeding, hypoxia, congenital heart disease, and antibiotic use, all of which contribute to an imbalance between pro- and anti-inflammatory mediators [2]. The early symptoms of NEC are often atypical, and the absence of a straightforward or precise diagnostic methods makes differentiation challenging, leading to potential oversight [3]. By the time infants present with more evident symptoms, such as vomiting, abdominal distension, diarrhea, and abdominal masses, the optimal window for timely medical intervention is frequently missed. Severe intestinal complications and long-term sequelae substantially worsen the prognosis of NEC [4]. Consequently, the identification of new therapeutic targets and drugs for NEC has become a key focus of current research.

Pyroptosis, a form of inflammatory cell death facilitated by the activation of caspases and subsequent cleavage of gasdermin family proteins, plays a pivotal role in the pathogenesis of acute and chronic diseases [5]. While moderate pyroptosis aids in host defense against pathogenic infections, excessive pyroptosis is linked to extensive cell death and severe tissue damage. In pathological states, it disrupts the intestinal mucosal barrier and dysregulates immune responses, thereby contributing to intestinal diseases [6]. Pyroptosis can be triggered by the canonical caspase-1 and non-canonical caspase-4 and -5/11 pathways [7], which cleave gasdermin D (GSDMD) into its N-terminal fragment (GSDMD-NT), facilitating pore formation. A strong association between pyroptosis and NEC occurrence and progression has been established [8]. The nucleotide-binding domain, leucine-rich-containing family, pyrin domain-containing-3 (NLRP3) inflammasome is activated in the intestinal tissues of children with NEC, and the inhibition of NLRP3 activity has been demonstrated to decrease intestinal inflammation and increase survival rates in NEC pups [9]. However, the genetic and molecular mechanisms underlying pyroptosis in NEC pathogenesis and sequelae remain unclear.

Bulk RNA sequencing analyzes a population of cells to obtain an average gene expression profile. In contrast, single-cell RNA sequencing (scRNA-seq) examines gene expression at the individual cell level, revealing cellular heterogeneity and offering deeper insights into the diversity and dynamics of cellular responses within tissue [10]. Therefore, we combined single-cell data analysis with transcriptomic data using bioinformatics methods to identify potential small molecules that target pyroptotic cell death mechanisms and to discover their therapeutic effects on NEC.

In the present study, we identified a population of highly inflammatory *TREM1^+^CD163^+^* macrophages associated with pyroptosis in the pathogenesis of NEC. We screened for ligand genes involved in cellular interactions by combining scRNA-seq and bulk transcriptome data. In addition, we identified the pyroptosis-related gene features and associated functional pathways. The role of *TREM1* in macrophages pyroptosis was validated in human intestine issue and cellular samples. This study advances our understanding of the mechanisms underlying pyroptosis in NEC and provides a foundation for future mechanistic studies to reveal critical pathogenic pathways and potential therapeutic targets.

## 2. Results

### 2.1. Clustering and Identification of Key Cell Subtypes at Single-Cell Resolution

**Figure 1:**
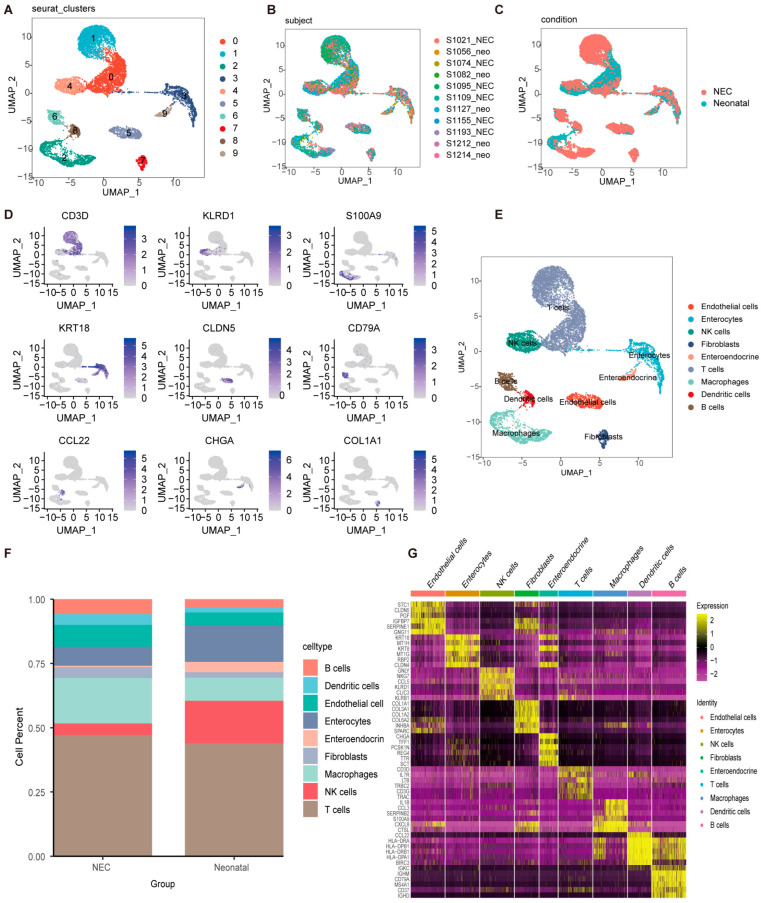
Clustering and distribution analysis of cell subpopulations. (**A**) UMAP dimensionality reduction revealed 10 distinct cell subpopulations. (**B**) UMAP plot showing the distribution of 12 samples. (**C**) UMAP plot showing the distribution of NEC and neonatal samples. (**D**) UMAP cluster map of the top marker gene for each cell subtype. (**E**) Nine cell subpopulations including B cells, dendritic cells, endothelial cells, enterocytes, enteroendocrine cells, fibroblasts, macrophages, NK cells, and T cells were annotated via the singleR package (version 2.8.0). (**F**) Histograms were performed to analyze cell type proportions. (**G**) The expression heatmap was used to show the top 6 marker genes in different cell subtypes.

Initially, the Seurat R package was used to filter low-quality cells. The UMI count significantly correlated with mRNA quantity, whereas the UMI/mRNA ratio was not significantly associated with mitochondrial gene quantity. Figure S1A,B depict violin plots before and after quality control, respectively. Subsequently, PCA was employed to normalize the data, and UMAP was conducted for dimensionality reduction and macrophage cell clustering. Nine subpopulations were identified and annotated using classical immune cell markers. The distinct subpopulations after clustering are presented in Figure 1A and the distribution of the 11 samples is summarized in Figure 1B. NEC and neonatal samples were also plotted (Figure 1C). Marker genes within the nine subpopulations were selected using the FindAllMarkers function. The distribution of the top marker genes across these subpopulations is shown in Figure 1D. Subsequently, nine cell subpopulations, namely B cells, dendritic cells, endothelial cells, enterocytes, enteroendocrine cells, fibroblasts, macrophages, NK cells, and T cells, were annotated (Figure 1E). Analysis of cell type proportions revealed a higher abundance of macrophages and lower abundance of natural killer (NK) cells in the NEC group compared to the neonatal samples (Figure 1F). The top six genes across different cell types are illustrated in Figure 1G. These findings indicate that macrophages are closely associated with pyroptosis and are crucial for its initiation and regulation.

### 2.2. Identification of DE-PRGs in NEC

**Figure 2:**
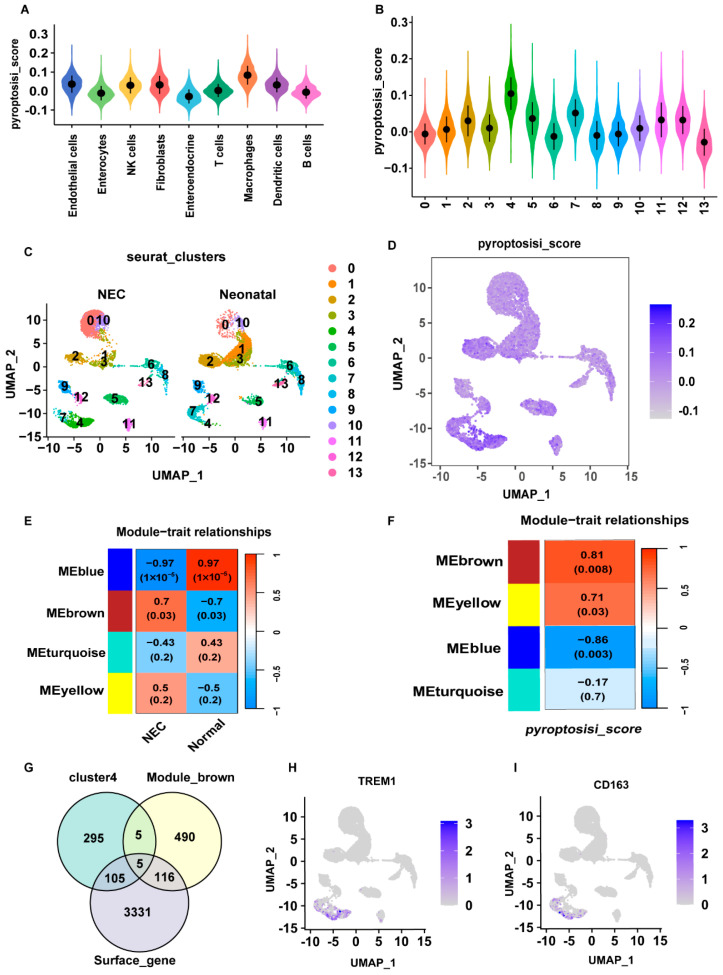
Analysis of pyroptosis-related gene expression and WGCNA results in NEC. (**A**) Violin plots were displayed to illustrate pyroptosis scores in different cell types. (**B**) Violin plots were displayed to illustrate pyroptosis scores in different cell clusters. (**C**) UMAP plot showing cell compositions between NEC and neonatal groups. (**D**) UMAP plot showing pyroptosis scores’ distribution of cell populations. (**E**) Heatmap of correlation of modes and phenotypes. (**F**) Heatmap of correlation of modules and pyroptosis scores. (**G**) Intersection of MEbrown module, cluster four, and surface genes in NEC and control. (**H**,**I**) Distribution of TREM1 and CD163 in the NEC group displayed by UMAP plot.

We performed WGCNA and pyroptosis scoring to identify key genes closely associated with pyroptosis. First, we downloaded the relevant gene sets to calculate pyroptosis scores and assess cellular pyroptosis (Table S1). Figure 2A illustrates the distribution of pyroptosis scores among the different immune cells, with macrophages exhibiting the highest scores compared to other immune cells. Moreover, we divided the cells into 13 clusters and calculated the pyroptosis scores using single-cell analysis. Of these, cluster 4 exhibited elevated pyroptosis scores (Figure 2B). scRNA-seq analysis conducted to compare the cell compositions of the NEC and neonatal groups revealed a considerable increase in the abundance of cluster 4 in the NEC group compared with that in neonatal controls (Figure 2C). Moreover, macrophage subtype cluster 4 exhibited a markedly higher pyroptosis score (Figure 2D). To select the modules and genes closely associated with NEC, we performed a WGCNA on the GSE46619 cohort. Dynamic tree-cutting algorithms yielded four modules, and module–trait correlation analysis indicated that the MEbrown module was more prevalent in the NEC group than in the neonatal group (Figure 2E) (Table S4). The MEbrown module also exhibited the highest pyroptosis score (Figure 2F). We identified five genes by overlapping the MEbrown module, cluster 4, and surface genes (Tables S3 and S5) (Figure 2G). Five genes, *TREM1, TLN1, NOTCH2, MPZL1*, and *ADA*, were identified as diagnostic markers of pyroptosis, with the top gene being *TREM1*. Considering that CD163 is highly expressed in macrophages, UMAP was used to display the distribution and expression of *TREM1* and *CD163* in the NEC group (Figure 2H,I). These results demonstrated that these five genes could accurately predict the disease status of NEC.

### 2.3. Interaction of Effector TREM1^+^CD163^+^ Macrophages with Other Cell Clusters

**Figure 3:**
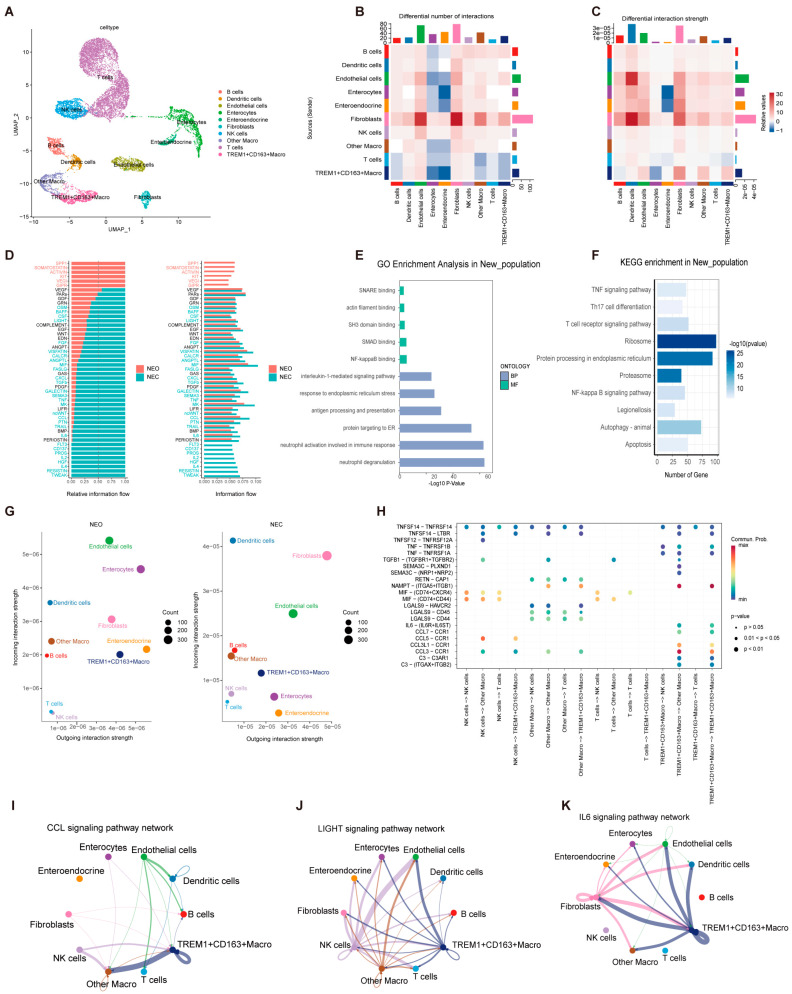
Characterization of *TREM1^+^CD163^+^* macrophages and interactions analysis. (**A**) Identification of *TREM1^+^CD163^+^* macrophages in the NEC group displayed by UMAP plot. (**B**,**C**) Heatmaps displayed the quantity and intensity of interactions between different cell types. (**D**) Signaling pathway information flow intensity between NEC and neonatal. (**E**) GO enrichment analysis in *TREM1^+^CD163^+^* macrophages. (**F**) KEGG enrichment analysis in *TREM1^+^CD163^+^* macrophages. (**G**) The scatter plot showed the number of interactions between the 10 cell clusters in NEC and neonatal (the *X*-axis represents the number of outgoing interactions and the *Y*-axis represents the number of incoming interactions). (**H**) The point diagram showed the receptor–ligand interactions among the cell clusters. (**I**–**K**) The network showed the interaction intensity between 10 cell clusters through the CCL, LIGHT, and IL-6 signaling pathway network.

To better explore the role of macrophage pyroptosis in NEC, we identified a new subtype of macrophages: *TREM1^+^CD163^+^* macrophages. Macrophages were then divided into two clusters according to the top hub gene (Figure 3A). Next, ligand–receptor interactions among the cells were explored using CellChat. We constructed a heatmap to elucidate the quantitative and qualitative dynamics of cell–cell interactions identified through single-cell analysis (Figure 3B,C). The results indicated that fibroblasts had the most extensive interactions with other cell groups, followed by endothelial cells. *TREM1^+^CD163^+^* macrophages showed a lower intensity and quantity of receptor activity than did the other macrophage groups. However, when acting as ligand cells, *TREM1^+^CD163^+^* macrophages exhibited a greater intensity and quantity of signaling than the other macrophage groups did. In addition, we calculated the interactions between signaling pathways in both the NEC and normal groups. The interaction strength of SPP1, OSTATIN, ACTIVIN, KIT, VEGI, and GIPR increased in the neonatal group. In contrast, the proportions of FLT3, CD137, PROS, IL2, HGF, IL4, RESISTIN, and TWEAK increased in the NEC group (Figure 3D). GO analysis revealed that *TREM1^+^CD163^+^* macrophages were associated with SMAD binding, NF-κB binding, neutrophil degranulation, ophil activation involved in the immune response, and protein targeting to the endoplasmic reticulum (Figure 3E). KEGG analysis revealed that ribosome, protein processing in the endoplasmic reticulum, and proteasome pathways were enriched in *TREM1^+^CD163^+^* macrophages (Figure 3F). Subsequently, the specific pathways involved in cell–cell communication were explored (Figure 3G). The bubble diagram revealed the probability of cellular interactions involving different ligands and receptors, with the NAMPT-ITGA5/ITGB1 pathway and CCL3/CCR1 pathways exhibiting a notably higher likelihood of interaction (Figure 3H). *TREM1^+^CD163^+^* macrophages communicated with other macrophages mainly via the CCL signaling pathway (Figure 3I). Additionally, *TREM1^+^CD163^+^* macrophages could communicate with endothelial cells and T cells through the LIGHT signaling pathway (Figure 3J). *TREM1^+^CD163^+^* macrophages primarily communicated with endothelial cells, dendritic cells, fibroblasts, and other macrophages via the IL-6 signaling pathway (Figure 3K).

### 2.4. Exploring the Molecular Mechanism of Pyroptosis Based on Hub Genes

**Figure 4:**
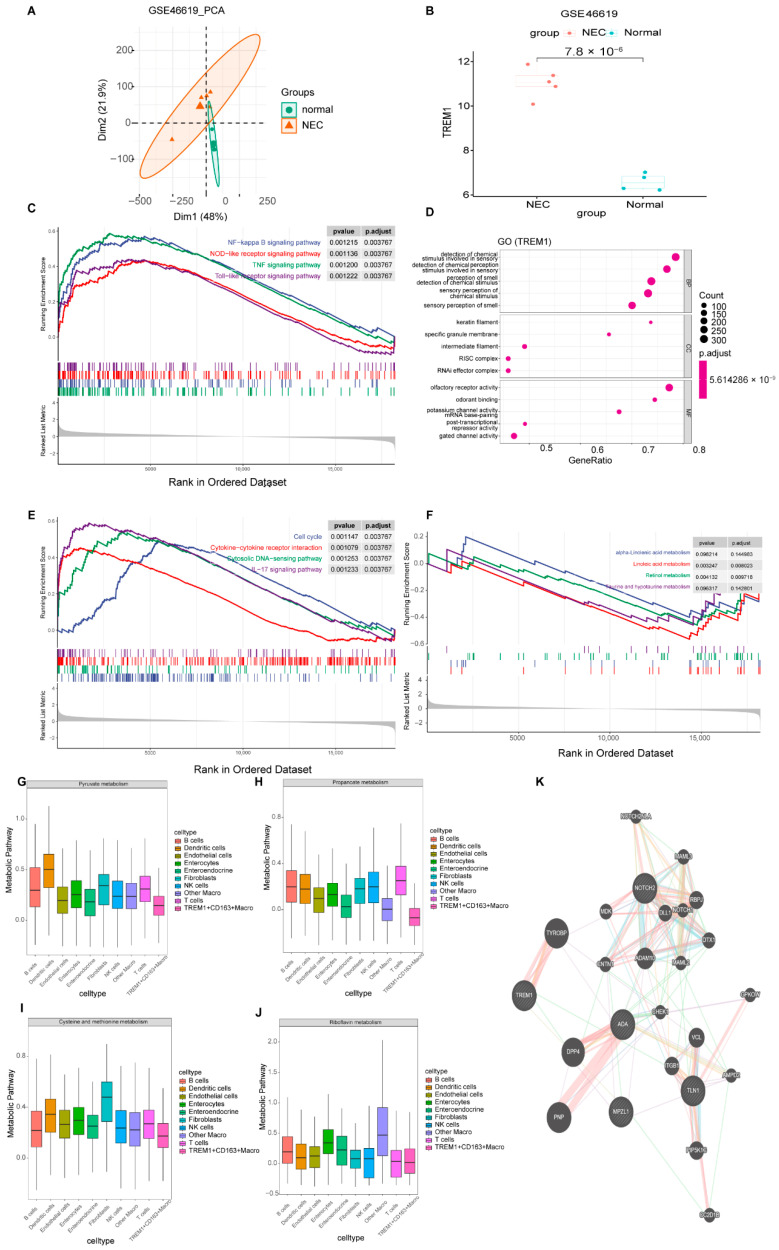
Identification of hub gene and GSEA and metabolism analysis in NEC and normal groups. (**A**) PCA of bulk RNA-seq data. (**B**) Box plots of feature gene expression between NEC and Control. (**C**) KEGG enrichment analysis in NEC. (**D**) GO enrichment analysis in NEC. (**E**,**F**) GSEA of metabolic processes between high-TREM1 and low-TREM1 groups. (**G**–**J**) Box plot visualizing the pyruvate metabolism, propanoate metabolism, cysteine and methionine metabolism, and riboflavin metabolism between 10 cell clusters. (**K**) PPI network of significantly differentially expressed pyroptosis-related genes.

To validate the role of TREM1 in pyroptosis, bulk RNA-seq data were used to explore gene expression. PCA demonstrated that the enrichment scores of each cell cluster effectively differentiated NEC samples from the control samples (Figure 4A). The expression of TREM1 was significantly higher in the NEC group than that in the normal group (Figure 4B). To analyze the impact of high and low TREM1 subgroups on NEC, we conducted GSEA using the ClusterProfile package. GO analysis indicated that the high TREM1 group was enriched in the following: olfactory receptor activity and gated channel activity (GO-molecular function terms); RISC complex and RNAi effector complex (GO-cellular component terms); and the detection of chemical stimulus involved in sensory and detection of chemical perception stimulus involved in sensory function (GO-biological process terms) (Figure 4D). The KEGG analysis demonstrated that the high TREM1 group was significantly enriched in the NF−κB signaling pathway, NOD-like receptor signaling, TNF signaling pathway, and Toll-like receptor signaling pathway, all of which are primarily associated with NEC pyroptosis (Figure 4C). To further evaluate the impact of TREM1 on cell proliferation and inflammatory pathways, the KEGG results indicated that the cell cycle, cytokine–cytokine receptor interaction, cytosolic DNA-sensing pathway, and IL−17 signaling pathway were enriched in the high TREM1 groups (Figure 4E). Metabolic disorders may influence intestinal development and function, thereby increasing the risk of developing NEC. KEGG enrichment analysis showed that TREM1 downregulated metabolic processes associated with NEC, including the metabolism of alpha-linolenic acid, linoleic acid, retinol, and taurine and hypotaurine (Figure 4F). Additionally, we analyzed the metabolism of *TREM1^+^CD163^+^* macrophages using scRNA data, and the results showed that the metabolism of pyruvate, propanoate, cysteine and methionine, and riboflavin was decreased in *TREM1^+^CD163^+^* macrophages, which was consistent with our bulk RNA analysis results (Figure 4G–J). Given that *TREM1*, *TLN1*, *NOTCH2*, *MPZL1*, and *ADA* were the five hub genes with the highest diagnostic efficacy, we constructed interaction networks using these genes and their associated counterparts. The strongest interaction was observed between *TREM1* and *TYROBP1*, suggesting a critical synergistic role in biological processes (Figure 4K).

### 2.5. Analysis of Hub Genes in the Human Intestine

**Figure 5:**
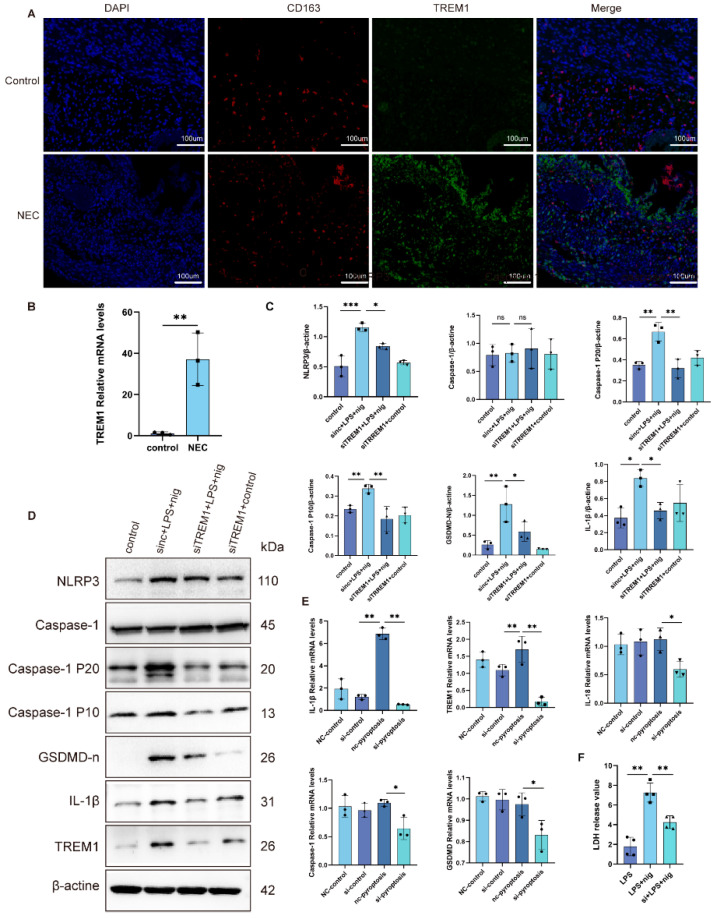
The results of experimental verification of the human intestine and cells. (**A**) Representative images of IF staining for CD163(red) and TREM1 (green). Scale bar = 100 μm. (**B**) mRNA expression of TREM1 in the control and NEC groups intestine (*n* = 3). (**C**,**D**) Western blotting analysis of TREM-1, NLRP3, caspase-1, caspase-1 P20, caspase-1 P10, GSDMD-n, and IL-1β (**E**) mRNA expression of TREM1, NLRP3, caspase-1, GSDMD, IL-1β, and IL-18. Data are shown as the means ± SD, * *p* < 0.05, ** *p* < 0.01, *** *p* < 0.001. (**F**) LDH release value of control, pyroptosis, and siTREM1pyroptosis groups.

We validated the mRNA expression of *TREM1* in the intestines of patients with NEC and neonates and found their elevated expression (Figure 5B). Protein levels were assessed using IF staining of intestinal tissue sections from patients with NEC and neonates. The levels of TREM1 and CD163 were elevated in patients with NEC compared to that of control patients (Figure 5A). These results corroborate the presence of *TREM1^+^CD163^+^* macrophages as a novel cell population in NEC that may play a critical role in disease heterogeneity.

### 2.6. Inhibition of TREM1 in THP-1 Cells Alleviates Cell Pyroptosis

To further investigate the mechanism of TREM1 in pyroptosis, we used THP-1 cells to construct a cell pyroptosis model, and siRNA to knockdown *TREM1* expression. Compared with the control, the expression of NLRP3, caspase-1, caspase-1 P10, caspase-1 P20, GSDMD-N, TREM1, and IL-1β were increased in the pyroptosis group, while the expression of these genes were partially restored after interference with TREM1 (Figure 5C,D). qRT-PCR results also showed that the expression of NLRP3-, caspase-1-, GSDMD-, TREM1-, and IL-1β-associated genes decreased after TREM1 was inhibited (Figure 5E). LDH release was used as an indicator of cell membrane integrity, and the results showed that knockdown of TREM1 significantly alleviated cell pyroptosis compared to the pyroptosis group (Figure 5F).

## 3. Discussion

Necrotizing enterocolitis is a common and severe inflammatory disease of the intestine in preterm infants [3]. Despite the continuous publication of relevant guidelines concerning its high incidence, high mortality rate, and rapid progression, it remains a significant challenge in clinical practice and a focal point for research. Cell death represents a fundamental biological process that manifests through distinct molecular pathways, with apoptosis and pyroptosis constituting two mechanistically and functionally divergent processes [4]. Apoptosis as a conserved programmed cell death mechanism operates as a non-inflammatory elimination system to remove superfluous or compromised cells. This process involves typical morphological changes such as cytoplasmic condensation, chromatin margination, and the formation of membrane-bound apoptotic bodies [5]. In contrast, pyroptosis, a novel form of programmed cell death, promotes the release of inflammatory cytokines IL-1β and IL-18, ultimately leading to a robust inflammatory response in the body [6]. This functional specialization shows the importance of pyroptosis in immunopathology and its therapeutic potential as a modulatable target in inflammatory disease treatments [7]. Increasing evidence suggests that pyroptosis is a key contributor to NEC [8]. Therefore, the inhibition of inflammatory signaling pathways associated with pyroptosis represents a promising therapeutic strategy for improving outcomes in patients with NEC [9,10]. However, the involvement of pyroptosis-related genes in NEC remains poorly understood.

We used scRNA-seq to analyze the heterogeneity of macrophages in NEC and explored the proportion of each cell type in the NEC and control groups [11]. Our analysis revealed that the number of macrophages was elevated in NEC, which prompted us to investigate their role in disease pathogenesis. Consistent with our findings, the results demonstrated that macrophages had the highest pyroptosis scores, suggesting that they may substantially contribute to cell pyroptosis and the pathophysiology of NEC. To better explore the role of macrophages in pyroptosis, we calculated pyroptosis scores among immune cells and found that cluster 4 cells belonging to the macrophage population were significantly enriched in NEC, exhibiting the highest pyroptosis scores.

To identified the hub genes in NEC, we conducted a comprehensive analysis of bulk RNA-seq. Then, WGCNA was using to identify an MEbrown module containing 616 shared genes associated with pyroptosis. We then identified five hub genes (*TREM1*, *TLN1*, *NOTCH2*, *MPZL1*, and *ADA*) from cluster 4, module genes, and surface genes, and further mapped them to the scRNA-seq data. While TREM1 is known to be closely associated with cell pyroptosis the roles of TLN1, NOTCH2, MPZL1, and ADA remain unclear [12,13]. TREM1 is a transmembrane immune receptor located on the surface of macrophages that amplifies the inflammatory response [14]. TREM1 enhanced myocardial pyroptosis in septic mice and activated the NLRP3 inflammasome in cardiomyocytes via the SMC4/NEMO pathway. Knowledge of this pathway may contribute to the treatment and prevention of septic cardiomyopathy [15]. Additionally, CD163 binds to the hemoglobin-haptoglobin complex, and its degradation is associated with antioxidant and anti-inflammatory effects [16]. Using CD163 to identify specific cell subpopulations, we identified *TREM1^+^CD163^+^* macrophages, which were specifically enriched in patients with NEC compared to control groups, this cell group exhibited pronounced pyroptosis.

Considering that *TREM1^+^CD163^+^* macrophage populations play a crucial role in the occurrence and progression of NEC, we further explored the cell–cell communication between *TREM1^+^CD163^+^* macrophage populations and other populations. Compared to other macrophages, *TREM1^+^CD163^+^* macrophages exhibited stronger signal reception than signal emission. Furthermore, *TREM1^+^CD163^+^* macrophages actively communicated with other cell groups and exhibited vigorous activity in the interaction of signaling pathways. GO analysis showed that *TREM1^+^CD163^+^* macrophages were associated with SMAD binding, NF-κB binding, neutrophil degranulation, and Phil activation was involved in immune response and protein targeting to the endoplasmic reticulum, which may have contributed to the cell differentiation and inflammatory response. KEGG analysis revealed significant enrichment in pathways related to protein processing in the endoplasmic reticulum and proteasome activity, which may influence the initiation and progression of NEC. Based on these results, it appears that *TREM1^+^CD163^+^* macrophages communicate with other macrophages via the NAMPT-ITGA5/ITGB1 and CCL3/CCR1 pathways. Recent studies have identified NAMPT as a newly recognized damage-associated molecular pattern that promotes NEC progression through dysregulated TGFβ and TLR4 signaling pathways [17]. Moreover, reduced NAMPT expression has been shown to reduce the mortality rate in neonatal mice by decreasing macrophage infiltration and differentiation [18]. Additionally, CCL3 is significantly overexpressed in NEC mice, exacerbating inflammatory bowel injury by modulating macrophage chemotaxis, which highlights CCL3 as a potential therapeutic target in NEC [19]. Yuan et al. demonstrated that the inhibition of CCL3 reduces intestinal tissue damage, while recombinant CCL3 aggravates intestinal tissue damage [20]. Furthermore, *TREM1^+^CD163^+^* macrophages communicate with other cell types through the LIGHT and IL6 signaling pathway networks, demonstrating their active participation in immune responses and inflammation regulation [21,22].

To further explore the role of TREM1 in NEC, we categorized the samples into two clusters based on mean TREM1 expression levels. GSEA conducted to investigate particular signaling pathways, including the NF−κB signaling pathway, NOD-like receptor, TNF signaling pathway, and Toll-like receptor signaling pathway associated with pyroptosis, exhibited high expression in samples with elevated TREM1. Moreover, the high TREM1 cluster was enriched in the cell cycle, cytokine–cytokine receptor interaction, cytosolic DNA sensing pathway, and IL−17 signaling pathway related to cell proliferation and inflammation.

Interaction networks based on TREM1, TLN1, NOTCH2, MPZL1, ADA, and their associated counterparts revealed strong interactions between TREM1 and TYROBP. TYROBP, also known as TYRO protein tyrosine kinase-binding protein, is a signaling adaptor protein expressed in cells involved in innate immune responses that mediates the activation of spleen tyrosine kinase [23]. The interaction between TREM1 and TYROBP (DAP12) constitutes a critical signaling axis in myeloid cells. Biochemical evidence confirms that TREM1 activation triggers TYROBP ITAM domain phosphorylation, initiating downstream PI3K and ERK pathway activation [24]. This signaling cascade promotes pro-inflammatory cytokine release while enhancing immune cell responsiveness, consistent with its established role in peripheral inflammation pathophysiology [25]. It is obvious that targeting components of the TREM-1/DAP12 pathway could be a promising therapeutic strategy for the treatment of inflammatory diseases [26]. Notably, our findings align with emerging reports implicating the TREM1-TYROBP complex in Alzheimer’s disease [27]. Recent studies indicate that TREM-1 and its signaling adapter DAP12 play a role in rheumatoid arthritis. Inhibiting DAP12 could effectively block the activation of TREM-1, thereby reducing inflammation and disease progression [28]. Research has shown that the TYROBP can induce the expression of the NLRP3 inflammasome and pyroptosis via sky signaling [29]. Based on our findings, we propose that elevated levels of TREM1 may interact with TYROBP to promote pyroptosis, thereby exacerbating the progression of NEC. In addition, TREM1 plays a crucial role in NEC heterogeneity and enhances pyroptosis. Metabolic activity is essential for NEC development [30]. We found that the metabolism of pyruvate, propanoate, and cysteine and methionine, and riboflavin decreased in *TREM1^+^CD163^+^* macrophages compared to other cell types. These changes may be closely related to cellular energy demands, antioxidant capacity, amino acid supply, and overall cellular functions, suggesting shifts in cellular functions, proliferation, survival ability, and response to environmental stress [31].

Our study demonstrated that TREM1 was highly expressed in macrophages and case cells from patients with NEC. To investigate the underlying mechanisms, we used intestinal samples from patients with NEC and THP-1 cells for validation. Activation of TREM-1 promotes the phosphorylation of NF-kB, which subsequently leads to the increased production and release of pro-IL-1β and NLRP3 [32]. Additionally, TREM1 inhibits necroptosis in cardiomyocytes [33], microglia [34], and pulmonary epithelial cells [35], thereby alleviating disease progression. In our study, we observed increased TREM1 expression in the intestines of patients with NEC, and the inhibition of TREM1 in THP-1 cells significantly reduced the production of cytokines and pyroptosis-related genes and proteins.

However, this study has some significant limitations. First, the small amount of transcriptome data analyzed might have affected the reliability and universality of our findings. Second, the regulatory mechanisms underlying the signature genes in NEC are not yet fully understood, leaving important questions unanswered. Future research stemming from this study should address these limitations.

## 4. Materials and Methods

### 4.1. Data Sources

The NEC single-cell dataset was downloaded from Zonod (GSE178088), a platform for sharing and preserving research data, software, and publications. This includes data from six patients with NEC and five non-patients. The bulk RNA-seq data of patients with NEC and neonatal patients were obtained from the Gene Expression Omnibus (GEO) database using the “GEOquery” R package (vesion 2.74.0). GSE46619 was used for further analysis. In addition, 105 pyroptosis-related genes were identified from the International Cancer Genome Consortium, The Cancer Genome Atlas, and GEO databases (Table S1).

### 4.2. Collection and Storage of the Intestinal Tissue

Neonatal and NEC intestinal tissue samples were obtained by IRB-approved surgical resection. The intestines were removed from the leading edge, away from the injured area. Consent was not required, as the samples were anonymized and collected under a discarded specimen protocol approved by the Capital Institute of Pediatrics Affiliated Children’s Hospital (Approval number: SHERLL2024035). For RNA extraction, tissue was washed with cold phosphate-buffered saline (PBS) and stored at −80 °C. For paraffin blocks, the intestine was fixed in formalin for 48 h, transferred to ethanol, and embedded in paraffin.

### 4.3. scRNA-seq Analysis

For the scRNA-seq dataset, we generated objects and filtered out low-quality cells using standard data preprocessing procedures. To ensure data quality, we excluded genes detected in fewer than three cells, retained only those with more than 200 counts, and filtered out cells with mitochondrial content exceeding 30%. The data were standardized by scaling the unique molecular identifier (UMI) counts to a factor of 10,000. After logarithmic transformation, the ScaleData function of the Seurat package (v4.0.2) was used for further normalization. Normalized and corrected data metrics were used for subsequent analyses. The top 20 variable genes were analyzed using principal component analysis (PCA), with the first 20 principal components maintained for visualization and grouping using Uniform Manifold Approximation and Projection (UMAP). This analysis identified 10 distinct cell clusters labeled 0–9, as shown in Figure 1. Seurat’s FindAllMarkers function was used to annotate the cell types and identify marker genes for each type.

### 4.4. Functional Enrichment Analysis

Gene Ontology (GO) and Kyoto Encyclopedia of Genes and Genomes (KEGG) analyses were performed to investigate the pathways and protein functions of the clustered cells. Gene Set Enrichment Analysis (GSEA) was conducted using the ClusterProfile package (version 4.12.6). Additionally, to explore the interactions between the five selected genes and their counterparts, a protein–protein interaction analysis was conducted.

### 4.5. Gene Set Variation Analysis

Gene Set Variation Analysis (GSVA) is an unsupervised analytical tool designed to evaluate gene set variations across different datasets. GSVA converts gene expression data into activity scores for gene sets using RNA-Seq and microarray analyses. The GSVA R package (version 2.0.7) was used to identify associated pathways. The cell pyroptosis gene set was downloaded to calculate pyroptosis scores. Additionally, the immune gene set, which included 28 immune cell types, was obtained from a previous study [1]. The correlation between TREM1 expression levels and immune cell infiltration scores was evaluated using Pearson’s correlation coefficient.

### 4.6. Bulk RNA-seq Data Validation

The Aft modules were transformed into an expression matrix, and the GSE49916 dataset was categorized into groups. The preprocessCore R package (version 1.18.0) was used for normalization, and PCA was performed to evaluate cell clusters and groupings. We identified the expression patterns of pyroptosis-related genes in both the disease and control groups, which were displayed in box plots using the limma package in R (version 3.62.2).

### 4.7. Cell–Cell Communication Analysis

The R toolkit CellChat (version 2.1.0), which employs network analysis and pattern recognition methods, was used to analyze cell–cell communication based on the scRNA-seq data. Specifically, the toolkit was used to predict the primary signaling inputs and outputs of cells, determine how these cells and signals coordinate their functions, and explore cell–cell communication.

### 4.8. scMetabolism Analysis

The R package scMetabolism (version 5.0.1), which simplifies the quantification of single-cell metabolic activity by using a single-line command, was applied to the scRNA-seq matrix to score metabolic gene signatures, with the goal of quantifying gene sets associated with metabolic pathways. We compiled these metabolic gene sets by integrating published datasets and manually curating the gene sets from the KEGG and REACTOME databases.

### 4.9. Weighted Gene Co-Expression Network Analysis

Weighted Gene Co-expression Network Analysis (WGCNA) was performed using the GSE46619 dataset to identify critical module genes associated with pyroptosis. Genes with low expression variance were filtered out, and the remaining genes were used to construct a co-expression network. Using the chosen β, an adjacency matrix was generated by calculating pairwise Pearson correlations between gene expression profiles. This was subsequently transformed into a topological overlap matrix to measure gene connectivity. The expression profiles of each module were obtained using computer-characterized genes and clinical features. Modules and genes showing enhanced pyroptosis were selected for further analysis.

### 4.10. Immunofluorescence Experiments

The intestinal sections were fixed in 4% paraformaldehyde for 15 min prior to immunofluorescence (IF) staining. After blocking nonspecific binding with 5% goat serum at 37 °C for 1 h, the sections were incubated overnight at 4 °C with primary antibodies: rabbit monoclonal anti-TREM1 (ab225861, 1:500, Abcam, Cambridge, UK) and mouse monoclonal anti-CD163 (68218-1-Ig, 1:400, Proteintech, Rosemont, IL, USA). The following day, the sections were incubated with secondary antibodies, including goat anti-rabbit IgG (SA00013-4, 1:200, Proteintech) and goat anti-mouse IgG (SA00013-1, 1:200, Proteintech) for 4 h at 37 °C in the dark. Nuclei were stained with DAPI (D820010, Beijing Solarbio Science & Technology, Beijing, China) at 37 °C for 15 min. IF images were captured using a confocal laser-scanning microscope (Leica, Wetzlar, Germany), with six random fields selected per sample for analysis.

### 4.11. Cell Model of Pyroptosis and TREM1 Small-Interfering RNA (siRNA) Transfection

THP-1 cells were used to construct an in vitro model of pyroptosis [2]. TREM1 siRNAs and the corresponding negative controls (si-NC negative control) of TREM1 were obtained from Sangon Biotech (Shanghai, China). The sequences were (for the negative control) as follows: 50-UUCUCCGAACGUG UCACGUTT-30 (sense chain), 50-ACGUGACACGUUCGGAGAATT30 (antisense chain); for TREM1 they were as follows: 50-CCGGUGUUCAAUAUUGUCAUU-30 (sense chain), 50-AAUGAUAAUGUUGAACACCGG-30 (antisense chain). THP-1 cells were plated in a cell dish (Corning Inc., Corning, NY, USA) with 1640 (11875093, Gibco, Waltham, MA, USA) and 10% FBS (13011-8611, Every Green, Hangzhou, China). All cells were cultured at 37 °C in a 5% CO_2_ atmosphere. Phorbol 12-myristate 13-acetate (P1585, Sigma-Aldrich, St. Louis, MO, USA) was used to stimulate the differentiation of THP-1 cells into macrophages. The negative control (si-Ctrl) and TREM1 siRNA were transfected into cells using CALNP™ RNAi regent following the manufacturer’s protocol. At 24 h after post-transfection, quantitative reverse transcription-PCR (qRT-PCR) was performed. Twelve hours later, the cells were treated with lipopolysaccharide (LPS, L4391, Sigma; 1 µg/mL) for 4 h and nigericin (HY-100381, MCE; 10 µM) for 1 h. Subsequently, Western blot analysis was conducted and lactate dehydrogenase (LDH) activity in the cell supernatant was measured using an LDH Cytotoxicity Assay Kit (C0016, Beyotime, Haimen, Jiangsu, China).

### 4.12. Western Blot for Protein Expression

Total protein from THP-1 cells was extracted with RIPA buffer containing 1% phenylmethylsulfonyl fluoride. Protein concentration was measured using the BCA assay. Equal amounts of protein (30 µg) from each sample were subjected to Western blot analysis. Proteins were separated by 15% sodium dodecyl sulfate-polyacrylamide gel electrophoresis and transferred to polyvinylidene fluoride membranes. After blocking with 5% non-fat milk for 2 h at 37 °C, the membranes were incubated overnight at 4 °C with the following primary antibodies: anti-NLRP3 (AG-20B-0014, 1 μg/mL, Adipogen, San Diego, CA, USA), RRID:AB_2490202), anti-caspase-1/p120/p10 (22915-1-AP, 1:8000, Proteintech RRID:AB_2876874), anti-GSDMD-N (ab215203, 1:1000, Abcam RRID:AB_215203), anti-IL-1β (A11369, 1:800, Abclonal, Woburn, MA, USA, RRID:AB_2534142), anti-IL-1β (A16288, 1:800, Abclonal Woburn, MA, USA, RRID:AB_2769945), and anti-β-actin (66009-1-Ig, 1:2000, Proteintech RRID:AB_2687938). After incubating with the appropriate secondary antibodies at 37 °C for 1.5 h, protein bands were visualized using enhanced chemiluminescence. The protein band was quantified with ImageJ1.8.0 software (RRID:SCR_003070).

### 4.13. Reverse Transcription Quantitative PCR for mRNA Expression

Total RNA was isolated from THP-1 cells and intestinal tissues using an RNA extraction kit (9767; Takara Bio, Kusatsu, Japan). RNA concentration was quantified using a NanoDrop One/OneC spectrophotometer (Thermo Fisher Scientific, Waltham, MA, USA). Reverse transcription of RNA into cDNA was performed using All-In-One 5X RT Master Mix (G590, ABM Biotechnology Co., Vancouver, BC, Canada). A 2 µL aliquot of cDNA from each sample was used for qPCR amplification with BlasTaq™ 2X qPCR MasterMix (G891, ABM Biotechnology Co.) on a QuantStudio™ 7 Flex System and the primer sequence in Table S2. Relative mRNA expression was performed by the 2^−ΔΔCq^ method, normalizing to β-actin, with each experiment conducted in triplicate.

### 4.14. Statistical Analysis

All data processing was performed using the R software (version 4.4.0). GraphPad Prism (version 9.5) was utilized for data analysis, with results presented as the mean ± standard deviation (SD). Comparisons between two groups were conducted using the *t*-test, whereas multiple comparisons were assessed using one-way ANOVA or Wilcoxon test. *p* < 0.05 is considered significant.

## 5. Concusions

In conclusion, we identified that *TREM1^+^CD163^+^* macrophages play a crucial role in pyroptosis during NEC progression. Our findings elucidated the biological functions and molecular mechanisms of TREM1, confirming its high expression in vivo and validating its pyroptosis-promoting effects in vitro. These insights enhance our understanding of the role of pyroptosis in NEC.

## Data Availability

scRNA-seq is available at Zonodo (https://doi.org/10.5281/zenodo.5813397) (accessed on 11 September 2024) (GSE178088). Bulk RNA-seq is available at Gene Expression Omnibus (https://www.ncbi.nlm.nih.gov/geo/) (accessed on 11 September 2024) (GSE46619). We would prefer research information to be emailed to other researchers upon their request.

## Supplementary Materials

The following supporting information can be downloaded at https://www.mdpi.com/article/10.3390/ijms26094036/s1.
