# Supplementary material for: Identification and Exploration of Pyroptosis-Related Genes in Macrophage Cells Reveal Necrotizing Enterocolitis Heterogeneity Through Single-Cell and Bulk-Sequencing
Source: Int J Mol Sci. 2025 Apr 24;26(9):4036. doi: 10.3390/ijms26094036 (PMC12071306; doi:10.3390/ijms26094036)
Supplement: Supplementary file 1 [file ijms-26-04036-s001.zip › Figure S1.pdf]

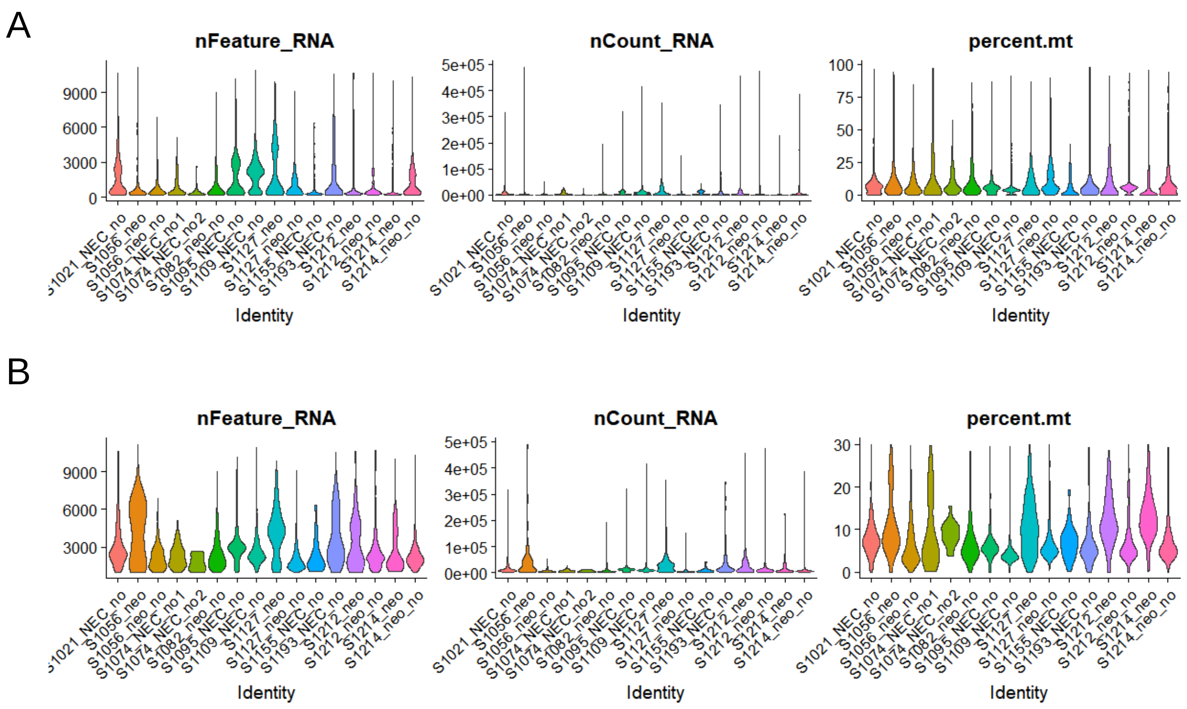

Figure S1. Gene Detection Distribution in Single-Cell Transcriptomic Data Before and After Quality Control

(A) Before Quality Control (B) After Quality Control The x-axis represents samples, feature represents genes, count represents gene counts, and percent represents the proportion of mitochondrial genes.
